# A multicentric study on understanding the bionomics of Indian malaria vectors across diverse eco-epidemiological settings

**DOI:** 10.1186/s13071-025-07159-2

**Published:** 2026-01-08

**Authors:** Ajeet Kumar Mohanty, Alex Eapen, Himmat Singh, Kuldeep Singh, Rajendra Kumar Baharia, Vidhan Jain, Debattam Mazumdar, Sachin Sharma, A. N. Shriram, P. T. Vidhya, Amit Sharma, Kannan Thiruvengadam, Manju Rahi

**Affiliations:** 1 Field Unit, ICMR-National Institute of Malaria Research, Panaji, Goa India; 2 Field Unit, ICMR-National Institute of Malaria Research, Chennai, Tamil Nadu India; 3 ICMR-National Institute of Malaria Research, Dwarka, New Delhi, India; 4 Field Unit, ICMR-National Institute of Malaria Research, Guwahati, Assam India; 5 Field Unit, ICMR-National Institute of Malaria Research, Nadiad, Gujarat India; 6 ICMR-National Institute of Research in Tribal Health, Jabalpur, Madhya Pradesh India; 7 ICMR-Vector Control Research Centre, Puducherry, India; 8 International Centre for Genetic Engineering and Biotechnology, New Delhi, India

**Keywords:** Malaria transmission, *Anopheles* vector, *Plasmodium* spp., Sporozoite rate, Insecticide susceptibility assay, Vector control

## Abstract

**Background:**

India aims to eliminate malaria by 2030; however, a thorough understanding of the current biology and behavior of vector species will facilitate the efforts. Vector species often alter their biting and resting behaviors in response to long-term chemical control measures, posing significant challenges to ongoing vector control interventions. Therefore, it is essential to investigate and update our knowledge of the bionomics of malaria vectors in the current context.

**Methods:**

This study was carried out across 14 districts in eight Indian states between 2021 and 2023, employing various entomological techniques. *Anopheles* mosquito species were tested for human blood meal preference and *Plasmodium* infection using polymerase chain reaction (PCR). Insecticide susceptibility status was assessed according to World Health Organization (WHO) protocols, and key metrics, such as degree of exophily, trap density, human biting rate (HBR), and man-hour density (MHD), were determined to understand mosquito abundance and behavior.

**Results:**

*Anopheles culicifacies*, a major malaria vector species, was found in all study states. The highest indoor MHD of this species was 11.95, recorded in the Kanker district of Chhattisgarh, whereas 27.16 was the highest outdoor MHD as observed in the Bareilly district of Uttar Pradesh. In Assam and Tripura, *Anopheles minimus* exhibited differential resting behavior, whereas *An. baimaii* was found to be exophilic in Kokrajhar, Udalguri, and South Tripura. *An. stephensi* showed endophilic behavior with an indoor MHD of 4.36 in Barmer. *An. minimus* exhibited high anthropophagic behavior, with a human blood index of 0.94 in South Tripura. A high sporozoite infection rate was observed in *An. baimaii* (5.88) compared with the other vector species. *An. culicifacies* was found to be resistant to alpha-cypermethrin (0.05%) in the Jagdalpur and Kanker districts, with possible resistance in Barmer study sites, and resistant to deltamethrin (0.05%) in Kanker, Surendranagar, and Dahod. *An. stephensi* showed resistance to multiple insecticides in the North Goa.

**Conclusions:**

*An. culicifacies* was prevalent in all eight study states, with a higher abundance in Kanker and Bareilly. Changes in the resting behavior of *An. minimus* in Tripura, and insecticide resistance that has developed in *An*. *culicifacies* and *An. stephensi* against pyrethroids poses a significant concern. The findings of this study will aid in implementing effective vector control strategies in India’s pre-elimination efforts against malaria.

**Graphical abstract:**

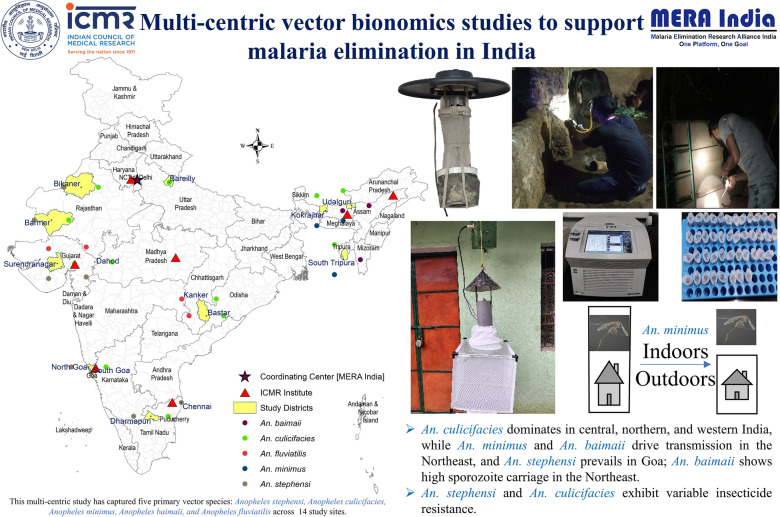

## Background

Malaria continues to be a significant vector-borne disease of public health concern, particularly in tropical and subtropical regions. Globally, in 2023, an estimated 263 million cases and 597,000 deaths occurred in 85 countries, resulting in a mortality rate of 13.7 deaths per 100,000 individuals at risk [1]. The World Health Organization (WHO) South-East Asia (SEA) region accounted for 1.5% of the global malaria cases, with India reporting half of all estimated cases in the region in 2023. Almost 48% of all estimated cases in the region were due to *Plasmodium vivax* [1]. According to the National Center for Vector Borne Diseases Control (NCVBDC), in 2023, 0.22 million cases of malaria were reported, reflecting an increase of ~30% from the number of cases in 2022 (0.17 million). In addition, 90% of the cases occurred in the states of Odisha, Jharkhand, Chhattisgarh, Uttar Pradesh, Maharashtra, Tripura, Mizoram, and West Bengal [2].

India has a diverse topography, with varied geo-climatic features. They range from tropical to subtropical, with different habitats, such as plains, wetlands, and mountains, which have resulted in a rich variety of flora, fauna, and ecosystems [3]. Habitat type also affects the diversity of malaria vectors and transmission risk. Thus, malaria is highly diverse across the country, with tribal and forested areas being the most endemic owing to a variety of ecological, biological, and sociocultural reasons [4–7].

In India, six Anopheline species are considered primary vectors for malaria transmission, including *An. stephensi*, *An. culicifacies*, *An. minimus*, *An. baimaii*, *An. fluviatilis*, and *An. sundaicus*. *An. culicifacies* and *An. fluviatilis* contribute to over 75–80% of malaria cases in the country [8, 9]. *An. culicifacies*, the most prevalent vector, is mostly found in plains, rural and periurban areas. *An. fluviatilis* is the abundant vector species in plateau and foothill areas. *An. minimus*, a perennial species, is an efficient vector in northeastern states, with a history of disappearance and reappearance [10–13]. *An. baimaii*, a part of *An. dirus* complex, is also a prevalent vector species in the northeastern states, often causing devastating disease outbreaks in conjunction with *An. minimus* [14–16]. *An. sundaicus* cytotype D is the only malaria vector in the Andaman and Nicobar Islands and is confined to these regions [17]. *An. stephensi* Liston is widely known as an urban malaria vector that breeds in domestic containers and is often associated with outbreaks in metropolitan cities [18–20]. In certain parts of the country, malaria transmission is sustained by secondary vectors such as *An. annularis*, *An. nivipes*, *An. philippinensis*, and *An. varuna* [9, 21].

India is committed to the elimination of malaria by 2030. With the launch of the National Framework for Malaria Elimination in 2016 and subsequently two national strategic plans (2017–2022) and (2023–2027) [2], India has strategized its elimination plans and categorized the states and districts on the basis of their endemic status. Malaria cases are heterogeneous in India across diverse geographical settings, primarily occurring in Madhya Pradesh, Jharkhand, Chhattisgarh, Maharashtra, Odisha, and the northeastern states [22, 23].

Since 2017, India has experienced a significant decline in malaria cases [24]. The decrease in malaria cases could be attributed to nationwide implementation of parasite and vector control strategies. Long-lasting insecticidal nets (LLINs) and indoor residual spraying (IRS) are the mainstay vector control strategies against malaria in India and other countries. Malathion and synthetic pyrethroids are predominantly used in IRS, while synthetic pyrethroids such as deltamethrin and alpha-cypermethrin are also used to impregnate bed nets [21, 23]. The complexity of malaria control strategies in India is heightened by the presence of various ecospecies and vector systems [25–27].

Entomological surveillance plays a crucial role in understanding the distribution, population density, change in behavior, and insecticide susceptibility status of vectors across the country. Such extensive surveillance assists in prioritizing control efforts spatiotemporally. It also provides essential information about the biological characteristics of vectors, which can help to evaluate the effectiveness of control interventions [28].

To enhance our understanding of the distribution and bionomics of malaria vectors in the context of epidemiological features in selected endemic districts of India, a multicentric study was carried out under the aegis of Malaria Elimination Research Alliance-India, a platform by the Indian Council of Medical Research (ICMR) to support operational research toward malaria elimination in India.

## Methods

### Study sites

**Fig. 1:**
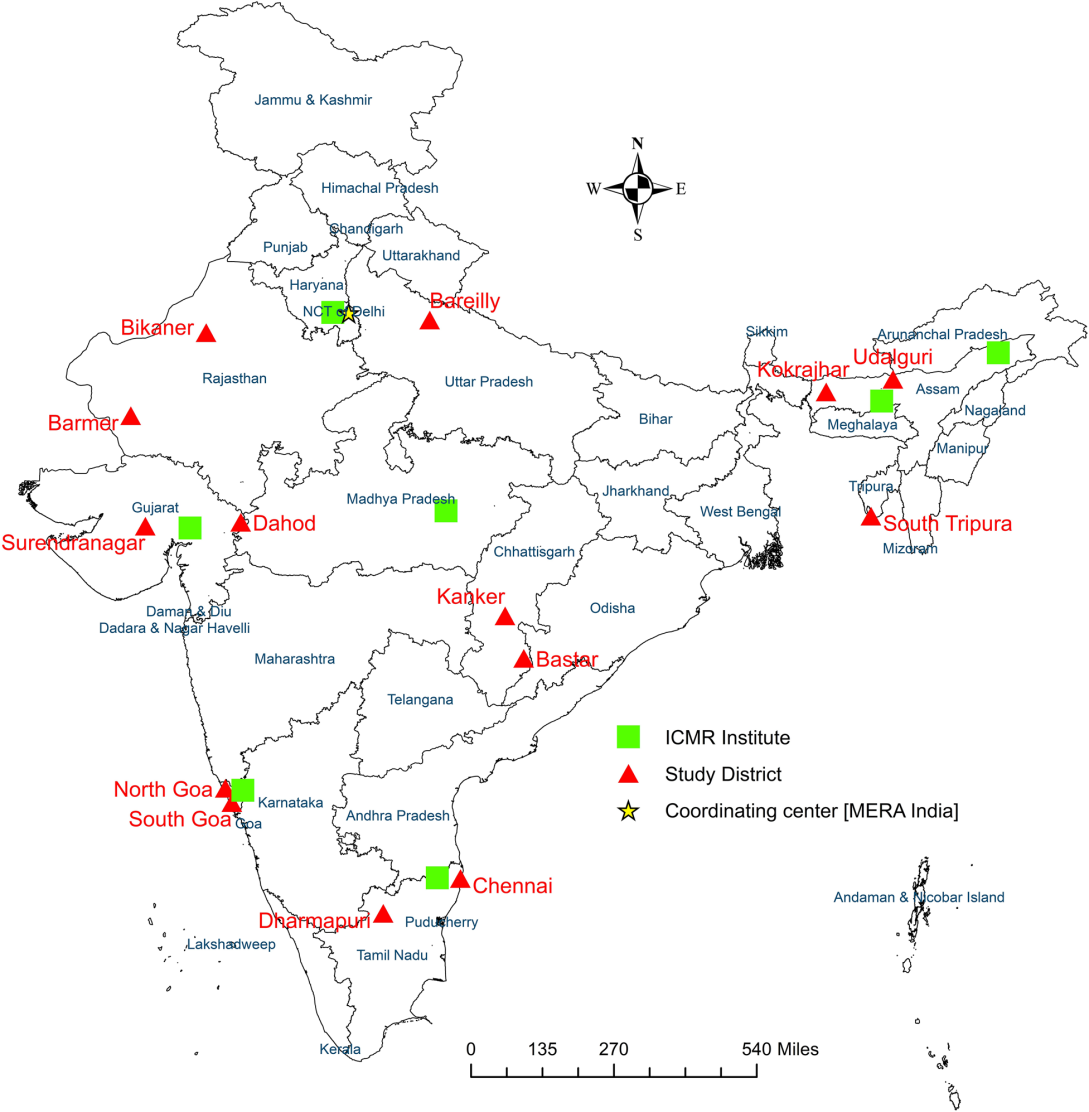
The locations of the study sites, encompassing 8 states and 14 districts across India, and investigating institutes of Indian Council of Medical Research (ICMR). The 14 districts were studied by the 2 institutes as follows: Assam, Goa, Gujarat, Rajasthan, Tamil Nadu and Uttar Pradesh were covered by NIMR and its field units; Chhattisgarh by NIRTH

The study was conducted in eight malaria-endemic states, namely Assam, Chhattisgarh, Goa, Gujarat, Rajasthan, Tamil Nadu, Tripura, and Uttar Pradesh, which reported different levels of endemicity and different malaria vectors. In total, 14 districts from these eight states were selected on the basis of their malaria endemicity and their location in distinct ecotypes. The study was carried out by two ICMR institutes: the National Institute of Malaria Research (NIMR, Delhi) and its field stations, which covered districts in the states of Assam, Goa, Gujarat, Rajasthan, Tamil Nadu, Tripura, and Uttar Pradesh, and the National Institute for Research in Tribal Health (NIRTH), Jabalpur, which covered Chhattisgarh study sites (Fig. 1).

Table 1 presents the topography of the study sites, their annual parasite incidence (API), and slide positivity rate (SPR), along with details of the malaria vectors and intervention methods reported for the selected districts in 2020, when the study was planned [2, 21]. In each district, six villages in rural areas or six wards in urban areas were selected in different malaria-endemic zones to represent various ecotypes, including plains, forests, foothills, and riverine across these eight study states. The seasonal collections were conducted in the Rajasthan and Tamil Nadu study states, whereas monthly collections were conducted in the remaining six states (Assam, Chhattisgarh, Goa, Gujarat, Tripura, and Uttar Pradesh).

**Table 1:** Epidemiological and entomological parameters of the study districts in 2020

S. no.	State	district	UHC/CHC/village/ward	Topography	API	SPR	Malaria vectors	Vector control intervention
1	Assam	Kokrajhar	Ultapani	Plain forest	0.11	0.14	Primary: *An. baimaii*, *An. minimus*, *An. culicifacies; *Secondary: *An. annularis*, *An. philippinensis/nivipes*, *An. aconitus*	DDT IRS, LLIN
		Udalguri	Orang and Udalguri	Plain forest, foothills	0.10	0.10		
2	Chhattisgarh	Bastar	Darbha and Bakawand	Forested and plain	4.91	7.02	Primary: *An. culicifacies*, *An. fluviatilis*	Alpha-cypermethrin IRS, LLIN
		Kanker	Durgkondal and Antagarh	Forested	2.87	2.41		
3	Goa	North Goa	Panaji, Candolim	Coastal plain, foothills	0.11	0.08	Primary: *An. stephensi. *Secondary: *An. subpictus*	Temephos-larvicide, LLIN
		South Goa	Madgaon, Cortalim	Coastal plain, foothills	0.03	0.04		
4	Gujarat	Surendranagar	Muli, Dharang Dhara	Plain	0.22	0.10	Primary: *An. culicifacies*	Alpha-cypermethrin IRS, LLIN
		Dahod	Dahod, Jhalod	Plain	0.04	0.01		
5	Rajasthan	Barmer	Piprali, Sonari, Gunga, and Kanaser	Plain	0.06	0.09	Primary: *An. culicifacies*, *An. stephensi*	Nil
		Bikaner	Diyatra, Bikampura, Champaser, and Kakra	Plain	0.00	0.00		
6	Tamil Nadu	Chennai	Royapuram	Plain	0.11	1.58	Primary: *An. culicifacies*, *An. stephensi*	Alpha-cypermethrin IRS
		Dharmapuri	Pennagaram	Plains, foothills	0.00	0.00		
7	Tripura	South Tripura	Purba Sambroom, East Luduwa, Magroom, Purba Laxmichara, Betaga, and Gobindabari	Plain	1.15	1.60	Primary: *An. baimaii. *Secondary: *An. annularis*	DDT IRS, LLIN
8	Uttar Pradesh	Bareilly	Majhgavan, Ramnagar, Bhmora, and Meerganj	Plain	2.22	13.10	Primary: *An. culicifacies*	DDT IRS

UHC - Urban Health Center; PHC - Primary Health Center; API - Annual Parasite Incidence; SPR - Slide Positivity Rate; DDT - Dichloro Diphenyl Trichloroethane; IRS - Indoor Residual Spray; LLIN - Long Lasting Insecticide Treated Net; S. no. - Serial Number

### Entomological surveillance

During entomological surveillance, *Anopheles* mosquitoes were obtained using different collection methods, including indoor and outdoor resting, indoor and outdoor light traps, pyrethrum spray sheets, and human landing collections.

### Indoor resting collections

Anophelines resting inside cattle sheds, human dwellings, and mixed dwellings (kachha/pucca with mud walls, brick walls, and thatched cemented roofs) were collected using either mechanical or mouth aspirators and torch lights during the morning hours (6 a.m. to 8 a.m.) for indoor resting behavior of vector (endophilic). In each village or urban ward, three to four houses and two to four cattle sheds were selected as sentinel and random sites for indoor resting collections once a month in ten study sites and seasonally in four study sites by employing two insect collectors spending 15 min in each structure. The mosquito density was calculated as the man-hour density (MHD) [29].

### Outdoor resting collections

Anophelines resting outdoors were collected with the help of torch and mouth aspirators during the early morning hours (6 a.m. to 8 a.m.) from vegetation such as bushes, grasses from rock hollows, tree bases, culverts, and under bridges for outdoor resting behavior of vector (exophilic). In each village/urban ward, three to four vegetations sites were searched for outdoor resting collections once a month by employing two insect collectors spending 15 min at each site. The outdoor MHD was also calculated.

### Light trap collections

Centers for Disease Control and Prevention (CDC) light traps were used to collect mosquitoes from both indoor and outdoor areas to study vector density. Traps were hung at a height of approximately 2 m indoors near the host or door and outdoors in open places away from the inhabitants of the village and urban area from dusk to dawn (6 p.m. to 6 a.m.). Vector density from trap collections was calculated as the number of mosquitoes captured per trap per night (PTD) indoors or outdoors. In each village or urban ward, three to four houses and one to two cattle sheds were selected to deploy light traps both indoors and outdoors once a month [10].

### Pyrethrum spray sheet collection (PSC)

Anophelines resting inside the houses were sampled during the daytime (6 a.m. to 8 a.m.). The method involved collecting indoor resting mosquitoes on white cotton cloth after knocking them down by spraying them with 2% pyrethrum extract in kerosene oil (1:19). Floors of a hut /house, including rooms, were completely covered with a white cotton cloth, and the pyrethrum solution was sprayed in the room. Precautions were taken to plug all holes/openings, and the inhabitants were advised to vacate the premises before spraying. The door was kept closed, and 10 min after spraying, the mosquitoes that were knocked down by the spray were collected from a white cloth spread on the floor. In each village or urban ward, two to three human dwellings were selected for the PSC. The mosquitoes were picked up and kept in Petri dishes lined with wet cotton or filter paper and transported to the laboratory for species identification, and their abdominal status was recorded [21].

### Human landing collections

All night human-landing collections from dusk to dawn (6 p.m. to 6 a.m.) were carried out after obtaining consent from a volunteer. Mosquitoes were collected while they landed on the human host or during the process of biting the volunteer. Care was taken to promptly collect mosquitoes to avoid biting. Mosquitoes were collected as they landed on human hosts (one indoor and one outdoor). Human landing collections were carried out both indoors and outdoors once per month in the selected house or huts. These collections were used to estimate the human-biting rate (HBR), calculated as the number of female mosquitoes landed per man per night [29].

### Laboratory analysis

#### Species identification and gonotrophic condition

Mosquitoes from all collections were identified to the species level using standard identification keys; examined for abdominal condition; classified as unfed or fully fed and semi or half gravid and gravid; and recorded accordingly [30, 31].

#### Host feeding preference

Field-caught mosquito samples obtained from different collection methods were analyzed using polymerase chain reaction (PCR) to determine the host feeding preference of human or bovine blood meal sources [32–34].

#### Parous rates

The parous rate was determined by dissecting mosquitoes from human landing collections, PSC, and resting collections and by examining ovarian tracheoles. Female mosquitoes were classified as nulliparous if tracheoles were coiled and parous if tracheoles were uncoiled [35].

#### Vector incrimination

*Anopheles* species collected (from all collection methods) and the recognized vector species were assayed for the presence of sporozoites of human malaria parasites in their salivary glands using the PCR technique [36].

#### Insecticide susceptibility tests

Adult mosquito vector species were tested for their susceptibility to different insecticides following WHO guidelines [37].

#### Data analysis

All data collected during the study were tabulated in Microsoft Excel and analyzed. The different metrics used for analyzing the data are presented below.

- (a) The mosquito density from trap collections was calculated as follows: mosquito trap density = number of mosquitoes captured per trap per night [10].
- (b) The proportion of individual species of mosquitoes resting inside the dwellings was estimated as the man-hour density (MHD). MHD is the number of mosquitoes collected by one person for 1 h and is calculated by considering the total number of mosquitoes (*n*), time spent in minutes (*t*), and number of persons involved in the collection (*p*). MHD = *n* × 60/*t* × *p* [38].
- (c) The human biting rate was calculated from the number of female mosquitoes that landed or attempted to bite per bait per night [39, 40].
- (d) The human blood index (HBI) was calculated based on the proportion of mosquitoes fed on human blood [40].
- (e) The degree of exophily (DE) was calculated as DE = 1 − (1 / (*F* / HGG) × 100, where *F* represents the number of fed mosquitoes and HGG is the total number of gravid and half-gravid mosquitoes collected by PSCs [41].
- (f) The sporozoite infection rate was calculated on the basis of the percentage of mosquito specimens positive for sporozoites in their salivary glands.
- (g) The percentage parity was calculated on the basis of the number of parous mosquitoes from all dissected mosquitoes collected using different sampling methods [42].
- (h) The insecticide susceptibility assay was performed using the WHO tube bioassay method [37]. The susceptibility/resistance status of field-caught vector mosquitoes was categorized according to WHO interpretation criteria: Less than 90% mortality indicates resistance, 90%–97% mortality indicates possible resistance, and 98%–100% mortality indicates susceptibility to the insecticide [37].

## Results

### Resting behavior of malaria vectors across eight study states

**Fig. 2:**
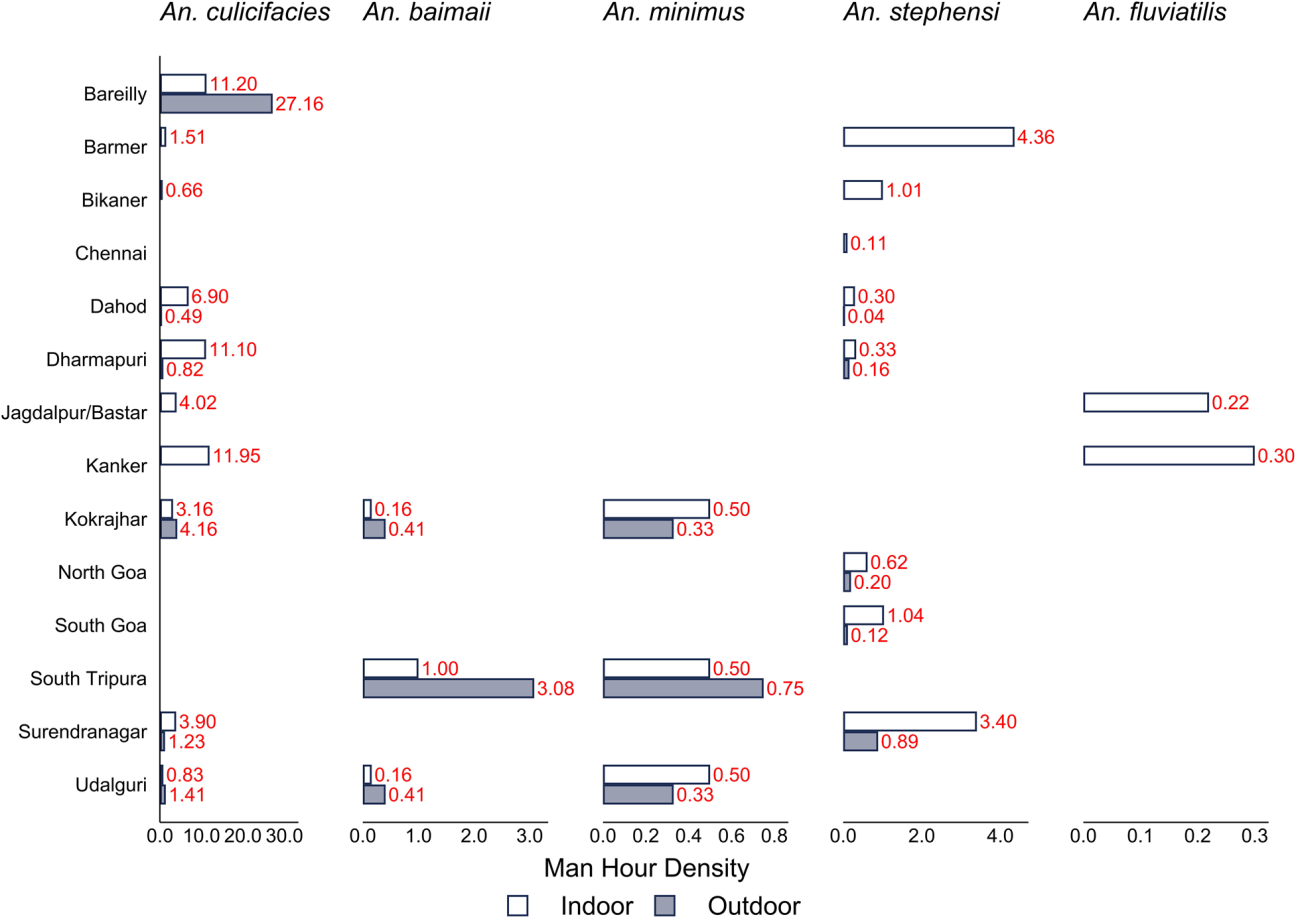
Man hour density (MHD) of malaria vectors collected in resting indoors and outdoors by hand catch method in the study sites

The MHDs of resting malaria vector species captured indoors and outdoors at all the study sites across eight states is presented in Table 2 and Fig. 2.

**Table 2:** Malaria vector density by various collection methods in study sites

S. No.	State	District	Species	Indoor resting collection (MHD)	Outdoor resting collection (MHD)	Light trap density indoor (PTD)	Light trap density outdoor (PTD)	Human biting rate (indoor) (HBR)	Human biting rate (outdoor) (HBR)
1	Assam	Kokrajhar	*An. culicifacies*	3.16	4.16	0.25	0.91	ND	ND
			*An. baimaii*	0.16	0.41	0.66	0.66	ND	ND
			*An. minimus*	0.50	0.33	0.91	1.58	ND	ND
		Udalguri	*An. culicifacies*	0.83	1.41	0.58	2.5	ND	ND
			*An. baimaii*	0.16	0.41	0.16	0.66	ND	ND
			*An. minimus*	0.50	0.33	0.50	1.58	ND	ND
2	Chhattisgarh	Jagdalpur/Bastar	*An. culicifacies*	4.02	ND	0.04	0.46	0	0
			*An. fluviatilis*	0.22	ND	0.21	0.13	0	0
		Kanker	*An. culicifacies*	11.95	ND	0.67	1.25	0	0
			*An. fluviatilis*	0.3	ND	0	0.42	0	0
3	Goa	North Goa	*An. stephensi*	0.62	0.20	0.08	0.08	1.48	1.66
			*An. culicifacies*	0	0	0	0.003	0	0.03
		South Goa	*An. stephensi*	1.04	0.12	0.14	0.12	2.65	2.75
4	Gujarat	Surendranagar	*An. culicifacies*	3.9	1.23	1.29	1.01	0.25	0.22
			*An. stephensi*	3.4	0.89	1.24	0.89	0.03	0.01
			*An. fluviatilis*	0	0	0.31	0.15	0	0
		Dahod	*An. culicifacies*	6.9	0.49	2.5	1.35	0.23	0.17
			*An. stephensi*	0.3	0.04	0.28	0.01	0	0
			*An. fluviatilis*	0	0	0.04	0.03	0	0
5	Rajasthan	Barmer	*An. culicifacies*	1.51	0	0.13	0.17	1	0
			*An. stephensi*	4.36	0	0.29	4.13	1	0
		Bikaner	*An. culicifacies*	0.66	0	0.08	0	1	0
			*An. stephensi*	1.01	0	0.38	1.33	1	0
6	Tamil Nadu	Chennai	*An. stephensi*	0.11	0	0.25	0	ND	ND
		Dharmapuri	*An. culicifacies*	11.1	0.82	3.25	0	ND	ND
			*An. stephensi*	0.33	0.16	0.25	0	ND	ND
7	Tripura	South Tripura	*An. culicifacies*	0	0	0.23	1.75	ND	ND
			*An .baimaii*	1.0	3.08	1.16	5.75	ND	ND
			*An. minimus*	0.5	0.75	3.33	4.58	ND	ND
8	Uttar Pradesh	Bareilly	*An. culicifacies*	11.2	27.16	3	5.5	ND	ND

MHD - Man Hour Density; PTD - Per Trap Density; HBR - Human Biting Rate; ND - Not Done; S. No. - Serial Number

#### Assam

In the Kokrajhar and Udalguri districts, three vector species, *An. culicifacies*, *An. baimaii*, and *An. minimus* were collected. Among these, *An. culicifacies* predominated with an indoor MHD of 3.16 in Kokrajhar and 0.83 in Udalguri. The indoor MHD of *An. minimus* (0.5) and *An. baimaii* (0.16) were the same in both districts. The outdoor MHD of *An. culicifacies* was higher in Kokrajhar (4.16) than in Udalguri (1.41). A similar outdoor MHD of *An. baimaii* (0.41) and *An. minimus* (0.33) were recorded in both districts.

#### Chhattisgarh

Jagdalpur and Kanker were the study sites where *An. culicifacies* and *An. fluviatilis* were captured from resting collections. The indoor MHD of *An. culicifacies* was higher than *An. fluviatilis*. A higher indoor MHD (11.95) of *An. culicifacies* was recorded in the Kanker district. *An. fluviatilis* was collected with an indoor MHD of 0.22 and 0.3 in Jagdalpur and Kanker, respectively.

#### Goa

The indoor MHD of *An. stephensi* was higher in South Goa (1.04) than in North Goa (0.62). The outdoor MHD was 0.20 in North Goa and 0.12 in South Goa.

#### Gujarat

Both the districts, Dahod and Surendranagar, recorded *An. culicifacies* and *An. stephensi*. In Surendranagar, the indoor MHDs of *An. culicifacies* and *An. stephensi* were 3.9 and 3.4, respectively, which were higher than the outdoor MHDs of 1.23 and 0.89. A similar trend for MHD was observed in Dahod district for these two vector species, indicating their endophilic nature. Furthermore, the MHD of *An. culicifacies* (indoor and outdoor) was higher than that of *An. stephensi* in both districts of Gujarat.

#### Rajasthan

In Rajasthan’s study districts, Barmer and Bikaner, the two main *Anopheles* species found in resting collections were *An. culicifacies* and *An. stephensi*. In Barmer, indoor MHD for *An. culicifacies* was 1.51, and in Bikaner, it was 0.66. For *An. stephensi*, indoor MHD was higher in Barmer (4.36) than in Bikaner (1.01). However, neither of the two vector species were captured outdoors, although the same efforts were made.

#### Tamil Nadu

Chennai and Dharmapuri were the two study sites in Tamil Nadu. In Chennai, *An. stephensi* was collected with an indoor MHD of 0.11, and in Dharmapuri, the MHD was 0.33. *An. culicifacies* was collected only in Dharmapuri, which had an indoor MHD of 11.1. The outdoor MHD in Dharmapuri was 0.82 for *An. culicifacies* and 0.16 for *An. stephensi*.

#### Tripura

In the South Tripura district, *An. baimaii* and *An. minimus* were found with indoor MHDs of 1.0 and 0.5, respectively. However, the outdoor resting density was higher, 3.08 for *An. baimaii* and 0.75 for *An. minimus*, indicating their exophilic behavior.

#### Uttar Pradesh

In the Bareilly district, *An. culicifacies* was the only vector species captured. The indoor and outdoor MHDs were 11.2 and 27.16, respectively.

In summary, across all 14 districts, Bareilly, Kokrajhar, and Udalgiri recorded a higher outdoor resting MHD for *An. culicifacies* compared with its indoor MHD, indicating its exophilic behavior. However, its endophilic behavior was recorded at the rest of the study sites (Table 2). The highest indoor MHD for *An. stephensi* was recorded in Barmer (4.36), whereas the lowest was recorded in Chennai (0.11). The highest outdoor MHD of this species was found in Surendranagar (0.89), and the lowest was in Dahod (0.04) (Fig. 2). Comparatively, both Chennai and Dharmapuri in Tamil Nadu exhibited lower abundances of *An. stephensi* during resting collections. Notably, no specimens of *An. stephensi* can be collected in the outdoors in Chennai, Barmer, and Bikaner because of its endophilic nature. Among the northeastern states, the outdoor MHD of *An. baimaii* was higher in Tripura and Assam, suggesting its exophilic nature.

### Comparison of the relative abundance of malaria vectors from light-trap collections

**Fig. 3:**
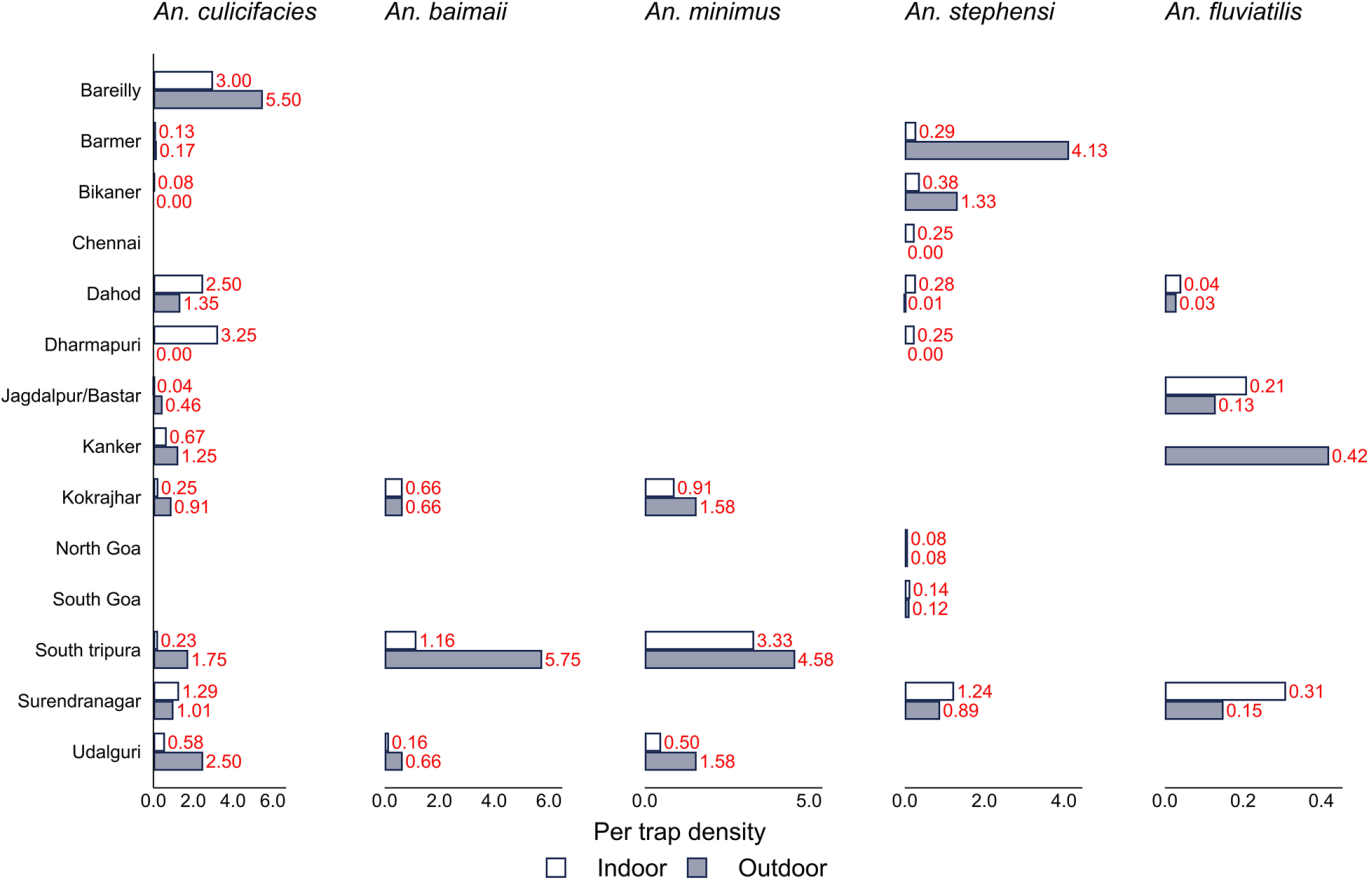
Per trap density (PTD) of malaria vectors collected by light traps in the study sites

The trap densities of malaria vectors in indoor and outdoor collections using light traps are shown in Fig. 3.

#### Assam

In the Kokrajhar district, the PTD of *An. minimus* was 0.91 indoors and 1.58 outdoors. In Udalguri, the corresponding values were 0.5 and 1.58. Both Kokrajhar and Udalguri districts recorded a higher per-trap density of *An. culicifacies* outdoors than indoors.

#### Chhattisgarh

In Jagdalpur and Kanker, the indoor PTDs of *An. culicifacies* were 0.04 and 0.67, respectively, which were lower than the outdoor PTD of Jagdalpur (0.46) and Kanker (1.25). *An. fluviatilis* had an indoor PTD of 0.21 in Jagdalpur, whereas this species was not found in the indoor trap collections in Kanker. However, the outdoor PTD of this species was 0.13 in Jagdalpur and 0.42 in Kanker.

#### Goa

In North Goa, the PTD of *An. stephensi* was 0.08 for both indoors and outdoors. In South Goa, the indoor PTD was 0.14, whereas outdoor density was 0.12, indicating a minor difference in their abundance.

#### Gujarat

The three malaria vector species *An. stephensi, An. culicifacies*, and *An. fluviatilis* were captured in Surendranagar and Dahod districts. *An. culicifacies* was found with a higher indoor PTD of 2.5 in the Dahod district than in Surendranagar (1.29). Similarly, for this species, the outdoor PTD was higher in Dahod (1.35) than in Surendranagar (1.01). *An. stephensi* and *An. fluviatilis* were found to have a higher indoor PTD than outdoor PTD in both districts. Overall, indoor densities were observed to be higher than outdoor densities for all three vector species at the Gujarat study sites.

#### Rajasthan

In Barmer, the indoor PTD of *An. culicifacies* was higher (0.13) than in Bikaner (0.08), while Barmer’s outdoor PTD was 0.17. For *An. stephensi*, the indoor PTD was 0.29 in Barmer and 0.38 in Bikaner, and the outdoor PTD was 4.13 in Barmer and 1.33 in Bikaner.

#### Tamil Nadu

Both Dharmapuri and Chennai recorded indoor PTD of 0.25 for *An. stephensi*. *An. culicifacies* was found only indoors in Dharmapuri, with a PTD of 3.25, whereas outdoor PTD remained at zero for both species.

#### Uttar Pradesh

In the Bareilly district, the indoor and outdoor PTDs of *An. culicifacies* were found to be 3 and 5.5, respectively. The outdoor PTD of this species was comparatively higher in Uttar Pradesh than at the other sites.

#### Tripura

In South Tripura, outdoor PTDs of *An. culicifacies*, *An. minimus*, and *An. baimaii* were 1.75, 4.58, and 5.75, respectively, which were higher than the indoor values.

In summary, the Dharmapuri district recorded the highest indoor PTD of *An. culicifacies* (3.25), whereas Bareilly recorded a higher outdoor PTD for this species (5.5). In addition, Surendranagar reported a higher indoor PTD for *An. stephensi* (1.24), whereas the indoor PTD of *An. stephensi* was lowest in North Goa (0.08). A higher outdoor PTD for *An. stephensi* was observed in Barmer (4.13), and a lower outdoor PTD was observed in Dahod (0.01). For *An. fluviatilis*, a higher indoor PTD was reported in Surendranagar (0.31), whereas the lowest was in Dahod (0.04). The higher outdoor PTD for *An. fluviatilis* was recorded in Kanker (0.42), and a lower PTD in Dahod (0.03). Overall, Tripura recorded higher indoor and outdoor PTD for *An. minimus* compared with Assam sites.

### Biting behavior of malaria vectors

The biting behavior of the vector species was studied using the human landing collection method in six districts (North Goa, South Goa, Surendranagar, Dahod, Barmer, and Bikaner) of three states (Goa, Gujarat, and Rajasthan). Human landing collection could not be conducted in the remaining eight districts of the five states (Assam, Chhattisgarh, Tamil Nadu, Tripura, and Uttar Pradesh) because of ethical challenges (Table 2). In Surendranagar district, the indoor human biting rate (HBR) of *An. culicifacies* was 0.25, whereas the outdoor HBR was 0.22. In Dahod, the indoor HBR for this species was 0.23, whereas it was 0.17 outdoors. In North Goa, the HBR of *An. stephensi* was 1.48 indoors and 1.66 outdoors, while a higher HBR was recorded in South Goa with values of 2.65 indoors and 2.75 outdoors (Table 2). In Barmer and Bikaner, the indoor HBR of *An. stephensi* and *An. culicifacies* was 1, whereas no vector species were captured outdoors.

Overall, the human biting rates of *An. stephensi* both indoors and outdoors were higher in the Goa districts (North and South Goa) than at the Gujarat study sites (Surendranagar and Dahod districts).

### Abdominal status of malaria vectors

The abdominal status of the different vector species captured from the pyrethrum spray sheet (PSC) collections and their degree of exophily at different study sites are presented in Table 3. Among the eight study states, data could not be collected for Assam, Goa, and Tripura because none of the vector species were captured during the PSC collections.

**Table 3:** Abdominal condition of the malaria vectors obtained from pyrethrum spray sheet collections at different study sites

S. no.	State	District	Species	Total	Fed (*F*)	Unfed	HG	G	HGG	*F*/HGG	*F*:HGG	DE = 1 − (1 / (*F* / HGG)) × 100
1	Gujarat	Surendranagar	*An. culicifacies*	552	325	15	77	135	212	325/212	1.53	34.77
			*An. fluviatilis*	7	6	1	0	0	0	6	6.00	83.33
			*An. stephensi*	291	173	41	51	26	77	173/77	2.25	55.49
		Dahod	*An. culicifacies*	802	456	18	143	185	328	456/328	1.39	28.07
			*An. stephensi*	23	13	7	3	0	3	13/3	4.33	76.92
2	Chattisgarh	Jagdalpur	*An. culicifacies*	21	16	4	1	0	1	16/1	16.00	93.75
		Kanker	*An. culicifacies*	46	33	8	3	2	5	33/5	6.60	84.85
3	Uttar Pradesh	Bareilly	*An. culicifacies *	286	174	19	30	63	93	174/93	1.87	46.55
4	Tamil Nadu	Dharmapuri	*An. culicifacies*	297	149	9	63	76	139	149/139	1.07	6.71
			*An. stephensi*	4	3	1	0	0	0	3	3.00	66.67

HG - Half gravid; G - Gravid; HGG - Half Gravid and Gravid; ND - Not Done; S. no. - Serial number

#### Chhattisgarh

A total of 67 *An. culicifacies* were collected from Jagdalpur and Kanker sites. Of these, 49 mosquitoes were found to be fed, while 6 were half gravid and gravid (HGG). A higher degree of exophily was observed for *An. culicifacies* in Jagdalpur (93.75%) and Kanker (84.85%).

#### Gujarat

To assess the abdominal status of the vector species, a total of 1675 Anopheline specimens were captured at the Gujarat sites. The vector species captured from the pyrethrum spray sheet collection were *An. culicifacies*, *An. stephensi*, and *An. fluviatilis*. Among these specimens, 973 were fed, 82 were unfed, and 620 were half gravid and gravid samples (HGG). *An. culicifacies* was the predominant species collected in Surendranagar and Dahod. *An. fluviatilis* exhibited a higher degree of exophily in the Surendranagar district (DE = 83.33). *An. stephensi* showed a higher degree of exophily in the Dahod district (DE = 76.92) than in Surendranagar (DE = 55.49). *An. culicifacies* exhibited a lower DE in both Surendranagar (DE = 34.77) and Dahod (DE = 28.07).

#### Tamil Nadu

At the Tamil Nadu sites, a total of 301 *An. culicifacies* and *An. stephensi* were captured from Dharmapuri using the PSC method. A higher degree of exophily was observed in *An. stephensi* (66.67) than *An. culicifacies* (6.71). However, there were no vector species captured at the Chennai sites.

To summarize, *An. culicifacies* exhibited a higher degree of exophily in Jagdalpur and Kanker districts (> 80%), and the degree of exophily was lower in Surendranagar, Dahod, Bareilly, and Dharmapuri districts (6.71%–34.77%). *An. stephensi* showed a higher degree of exophily in Dahod and Dharmapuri than in Surendranagar.

### Parity status of malaria vectors

**Fig. 4:**
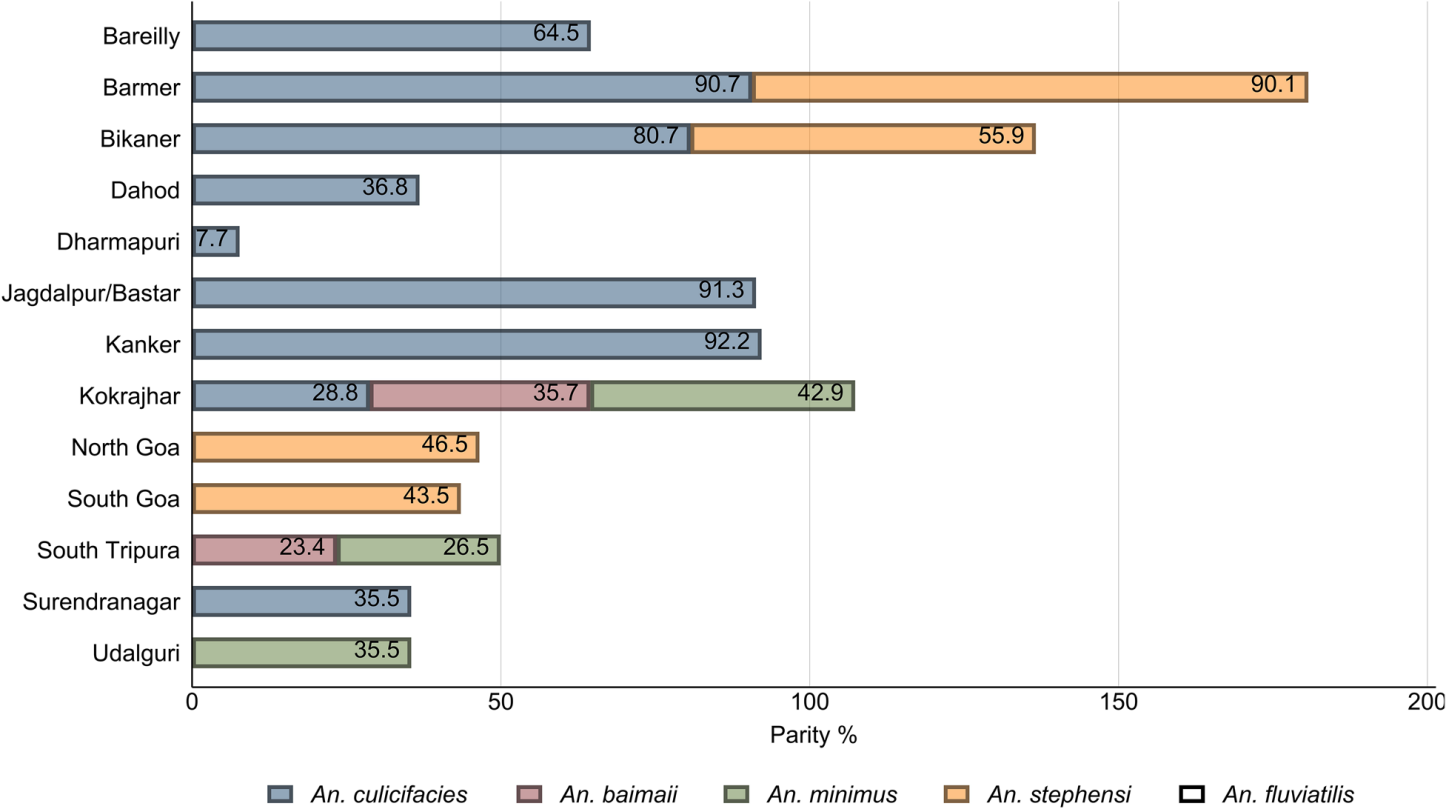
Parity status of malaria vectors obtained from different collection methods in the study sites

The parous rate, which is an indicator of longevity of malaria vector species, was determined from the study areas (Fig. 4). *An. culicifacies* showed a higher parous rate in Chhattisgarh’s Kanker (92.2%) and Jagdalpur (91.3%) sites, Rajasthan’s Barmer (90.7%) and Bikaner (80.7%) districts, followed by Uttar Pradesh’s Bareilly (64.5%) district. The parous rate of the species was lower in Gujarat’s Dahod (36.8%) and Surendranagar (35.5%) districts and Assam’s Kokrajhar (28.8%) district, and the lowest in Tamil Nadu’s Dharmapuri (7.7%) district. The parous rate of *An. stephensi* was highest in Rajasthan’s Barmer (90.1%) district followed by Bikaner (55.9%), North Goa (46.5%), and South Goa (43.5%) districts. Proportion of parous *An. baimaii* was highest in Kokrajhar (35.7%) followed by South Tripura (23.4%). The highest parous rate of *An. minimus* was recorded in Kokrajhar (42.9%) followed by Udalguri (35.5%) and South Tripura (26.5%).

In summary, the highest parous rate of *An. culicifacies* recorded in Jagdalpur and Kanker sites of Chhattisgarh and Barmer and Bikaner districts of Rajasthan (> 80%) might be owing to the favorable climatic conditions in these areas. Similar results were also observed for *An. stephensi* at the Rajasthan study sites.

### Anthropophagic behavior of malaria vectors

**Fig. 5:**
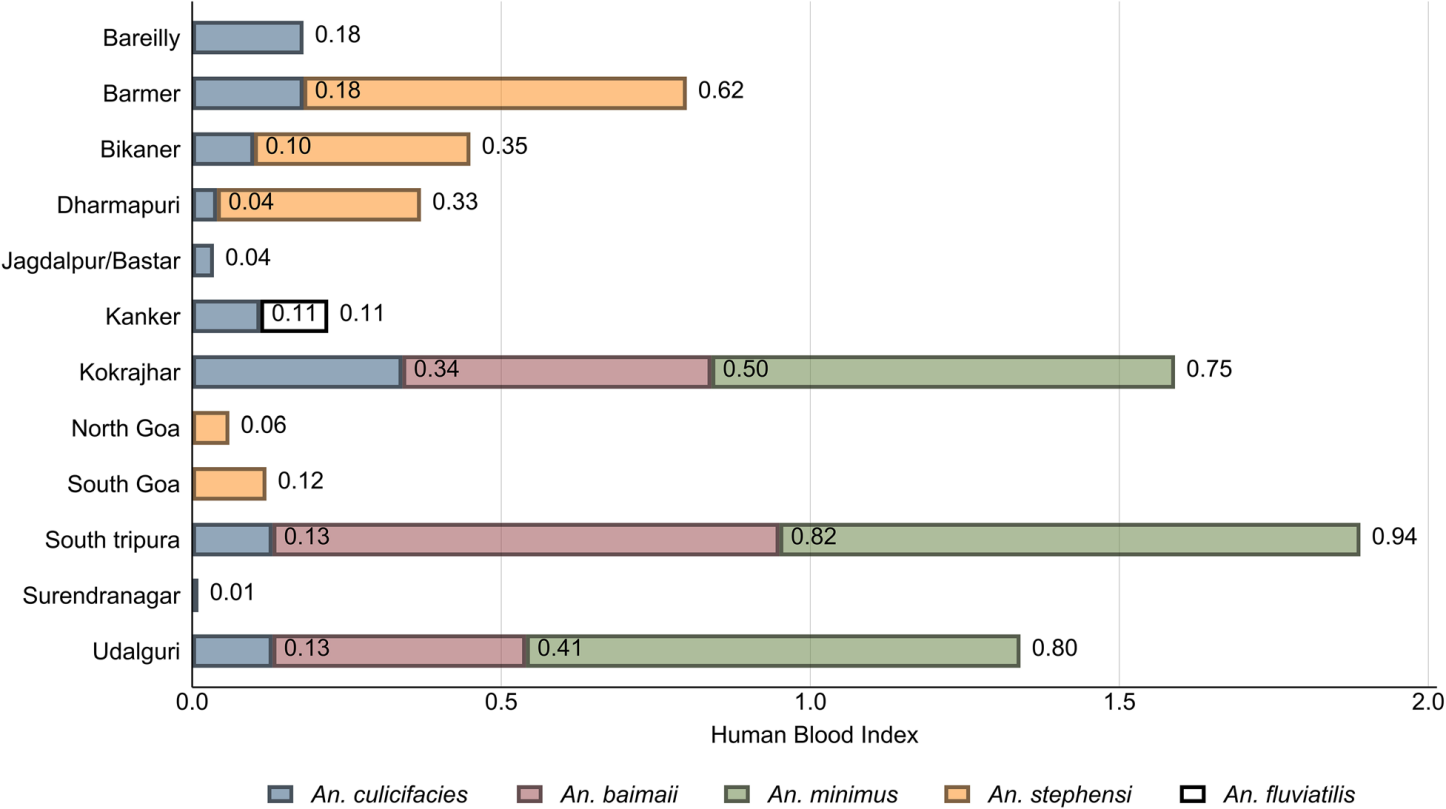
Human Blood Index (HBI) of malaria vectors collected by different collection methods in study sites

The blood meal preferences of the different malaria vector species at the study sites are shown in Fig. 5. The higher human blood index (HBI) of *An. culicifacies* was observed in Kokrajhar (0.34), and it was lower at the Surendranagar (0.01) study site. *An. stephensi* showed a higher HBI in Barmer (0.62) and a lower HBI in North Goa (0.06). The higher HBI of *An. minimus* was (0.94) in South Tripura and lower in Kokrajhar (0.75), whereas the HBI of *An. baimaii* was higher in South Tripura (0.82) than in Udalguri (0.41). Both *An. culicifacies* and *An. fluviatilis* were found with an HBI of 0.11 in the Kanker district.

Overall, there was a variation in HBI values observed between the vector species, namely, *An. stephensi*, *An. culicifacies*, *An. minimus*, and *An. baimaii*, indicating differential anthropophagic behavior across the study sites.

### *Plasmodium *infection rates of malaria vectors

The *Plasmodium* infection rates of malaria vectors obtained using different collection methods across the study sites are presented in Table 4. *An. culicifacies* was tested positive for human *Plasmodium* infection at four study districts: Dahod, Surendranagar, Kanker, and Bareilly. The sporozoite rates at these four districts were 1, 0.5, 0.32, and 0.29, respectively. Although *An. culicifacies* was collected from Jagdalpur, Barmer, Bikaner, Dharmapuri, Kokrajhar, Udalguri, and South Tripura, none tested positive for *Plasmodium* infection.

**Table 4:** *Plasmodium *infection rates of malaria vectors collected at different study sites

S. no.	State	District	Species	No. tested	No. positive	Pf positive	Pv positive	Sporozoite rate (%)
1	Assam	Kokrajhar	*An. culicifacies *	149	0	0	0	0
			*An. baimaii *	18	0	0	0	0
			*An. minimus*	26	1	1	–	3.85
		Udalguri	*An. culicifacies *	248	0	0	0	0
			*An. baimaii *	57	0	0	0	0
			*An. minimus*	76	1	1	–	1.32
2	Chhattisgarh	Jagdalpur/Bastar	*An. culicifacies*	159	0	0	0	0
			*An. fluviatilis*	17	0	0	0	0
		Kanker	*An. culicifacies*	623	2	2	–	0.32
			*An. fluviatilis*	23	0	0	0	0.0
3	Goa	North Goa	*An. stephensi*	57	1	0	1	1.75
		South Goa	*An. stephensi*	91	1	1	0	1.1
4	Gujarat	Surendranagar	*An. culicifacies*	200	1	1	–	0.5
		Dahod	*An. culicifacies*	200	2	1	1	1.0
5	Rajasthan	Barmer	*An. culicifacies*	126	0	0	0	0
		Bikaner	*An. culicifacies*	206	0	0	0	0
6	Tamil Nadu	Chennai	*An. stephensi*	40	0	0	0	0
		Dharmapuri	*An. culicifacies*	448	0	0	0	0
			*An. stephensi*	14	0	0	0	0
7	Tripura	South Tripura	*An. culicifacies*	21	0	0	0	0
			*An. baimaii*	34	2	2	–	5.88
			*An. minimus*	162	7	3	4	4.32
8	Uttar Pradesh	Bareilly	*An. culicifacies*	678	2	1	1	0.29

*Pv - Plasmodium vivax, Pf - Plasmodium falciparum*

*An. stephensi* tested positive for *Plasmodium* infection in both North and South Goa districts, with an average positivity rate of 1.43%. North Goa recorded a higher sporozoite positivity rate (1.75%) than South Goa (1.1%).

None of the *An. fluviatilis* samples tested positive for *Plasmodium* infection at all study sites. *An. minimus*, a malaria vector in the northeastern states, was found to be positive for *Plasmodium* infection in both Assam and Tripura, with a higher sporozoite rate in Tripura (4.32%) than in Assam (3.85%). Notably, *An. baimaii* collected from the South Tripura district showed a higher sporozoite rate (5.88%) among all the vector species tested, indicating a high vectorial role in the transmission of *P. falciparum*.

### Susceptibility status of malaria vectors to insecticides

The vector susceptibility assay was performed at 10 out of 14 study sites, namely, Jagdalpur, Kanker, North Goa, Surendranagar, Dahod, Bareilly, Barmer, Bikaner, Udalguri, and South Tripura (Table 5), and at the remaining 4 study sites, Chennai, Dharmapuri, Kokrajhar, and South Goa, the assay could not be performed because of the unavailability of sufficient numbers of vector species. *An. stephensi*, *An. culicifacies*, and *An. minimus* were tested for their susceptibility to different insecticides such as malathion, DDT, and synthetic pyrethroids, including deltamethrin, alpha-cypermethrin, and cyfluthrin, following WHO guidelines [21].

**Table 5:** Insecticide susceptibility status^*^ of malaria vectors at the study states in India

S. no.	State	District	Vector name	Alpha-cypermethrin 0.05%	Deltamethrin 0.05%	DDT 4%	Malathion 5%	Cyfluthrin 0.15%	Etofenprox 0.50%
1	Assam	Udalguri	*An. minimus*	98%	ND	ND	98%	98%	ND
2	Chattisgarh	Jagdalpur/Bastar	*An. culicifacies*	82.5%	91.3%	16.3%	75.0%	ND	ND
		Kanker	*An. culicifacies*	72.9%	69.9%	44.4%	63.5%	ND	ND
3	Goa	North Goa	*An. stephensi*	80%	93%	25%	33%	89%	99%
4	Gujarat	Surendranagar	*An. culicifacies*	99%	88%	ND	ND	76%	72%
			*An. stephensi*	99%	ND	ND	ND	ND	76%
		Dahod	*An. culicifacies*	98%	87%	ND	ND	72%	80%
			*An. stephensi*	99%	ND	ND	ND	ND	64%
5	Rajasthan	Barmer	*An. culicifacies*	97%	99.2%	46.8%	87%	ND	ND
			*An. stephensi*	96%	91%	66%	62%	ND	ND
		Bikaner	*An. culicifacies*	98%	100%	100%	100%	ND	ND
			*An. stephensi*	97%	94%	68%	76%	ND	ND
6	Uttar Pradesh	Bareilly	*An. culicifacies *	99.17%	99.09%	76.85%	71.84%	ND	ND
7	Tripura	South Tripura	*An. culicifacies *	100%	99.17%	67.5%	62.73%	ND	ND

^*^WHO interpretation criteria: 98–100% mortality = susceptible; 90–97% mortality = possible resistance; < 90% mortality = resistant; ND - not done

### Alpha-cypermethrin (0.05%)

In Chhattisgarh, *An. culicifacies* was resistant to alpha-cypermethrin in both the Jagdalpur and Kanker sites, with mortality rates of 82.5% and 72.9%, respectively. In contrast, *An. culicifacies* was found to be susceptible to alpha-cypermethrin in Surendranagar, Dahod, Bikaner, Bareilly, and South Tripura, whereas there was possible resistance observed in Barmer. Furthermore, *An. stephensi* showed resistance to alpha-cypermethrin in the North Goa district, whereas it was susceptible in Surendranagar and Dahod, and under a possible resistance category in Barmer and Bikaner. *An. minimus* remained susceptible to alpha-cypermethrin in Udalguri (Assam) (Table 5).

### Deltamethrin (0.05%)

*An. culicifacies* was found resistant to deltamethrin in both Gujarat districts. In Chhattisgarh, this species was possibly resistant to deltamethrin in Jagdalpur with 91.3% mortality, whereas in Kanker, it developed resistance with a mortality rate of 69.9%. *An. stephensi* showed possible resistance to deltamethrin in North Goa, Barmer, and Bikaner districts. In contrast, *An. culicifacies* was found susceptible to deltamethrin in Barmer, Bikaner, Bareilly, and South Tripura.

### DDT (4%)

*An. culicifacies* was resistant to DDT at the Jagdalpur, Kanker, Barmer, Bareilly, and South Tripura study sites. However, it was found to be susceptible to DDT in Bikaner. *An. stephensi* showed resistance to DDT at the North Goa, Barmer, and Bikaner study sites.

### Malathion (5%)

The malathion resistance in *An. culicifacies* was recorded at the Jagdalpur, Kanker, Barmer, Bareilly, and South Tripura study sites. However, *An. culicifacies* and *An. minimus* were susceptible to this insecticide in Bikaner and Udalguri. *An. stephensi* developed resistance to malathion in North Goa, Barmer, and Bikaner.

### Cyfluthrin (0.15%)

In North Goa, *An. stephensi* was resistant to cyfluthrin, whereas in the Surendranagar and Dahod districts, *An. culicifacies* also showed resistance to this insecticide. However, *An. minimus* was found to be susceptible to cyfluthrin in Udalguri (Assam state).

### Etofenprox (0.50%)

*An. stephensi* and *An. culicifacies* were resistant to etofenprox in the Surendranagar and Dahod districts, whereas *An. stephensi* was found to be susceptible in North Goa district.

In summary, *An. culicifacies* was found to be resistant to alpha-cypermethrin (0.05%) in two sites of Chhattisgarh (Jagdalpur and Kanker). The mosquito species was also resistant to deltamethrin (0.05%) in Kanker district of Chhattisgarh, two districts of Gujarat (Surendranagar and Dahod).

However, *An. culicifacies* remained susceptible to deltamethrin at the Rajasthan, Uttar Pradesh, and Tripura study sites. *An. culicifacies* was susceptible to DDT and malathion in Bikaner, whereas *An. stephensi* and *An. culicifacies* were resistant to these insecticides in the remaining areas.

## Discussion

India is making significant progress toward its goal of eliminating malaria by 2030. Understanding the current bionomics of malaria vectors is essential, especially as climate change and rapid urbanization impact the distribution, abundance, and behavior of different vector species [43–45]. Malaria is a heterogeneous communicable disease, and vector management strategies must be tailored based on vector bionomics in a specific region. Inadequate knowledge of the bionomics of primary vector species across the country may impede efforts to achieve malaria elimination [23].

Mosquitoes tend to alter their feeding and resting behaviors in response to chemical-based control interventions, creating significant challenges for the current vector control methods. Continuous exposure to insecticides causes selection pressure in mosquitoes and inevitably results in new forms of genetic or behavioral resistance [46]. In addition, it is crucial to update the current status of major vector species in response to changes in insecticide treatments [8, 47, 48].

The current bionomics study was conducted in 14 districts across eight states, including two northeastern states (Assam and Tripura), considering the diverse ecological setups of the country to address the knowledge gap regarding the biting behavior, resting patterns, and *Plasmodium* infection rate of the primary vectors.

*An. culicifacies*, *An. stephensi*, *An. fluviatilis*, *An. minimus*, and *An. baimaii* were the major malaria vectors encountered during the study, and their population densities were assessed using different collection methods (Table 2). The mosquito collection data revealed the presence of *An. culicifacies* at all study sites across the eight states, with a relatively higher density in rural areas. It is particularly abundant in rural tribal and semiurban ecological settings, indicating access to diverse hosts for blood meal [49]. A recent study (study period 2017–2020) reported that *An. culicifacies* could not be collected in the South Tripura district of Tripura [29]. However, the current study collected a considerable number of *An. culicifacies* by using light traps in Tripura. *An. culicifacies* was not a prominent vector species previously found in this region [50]. The ecological succession of this mosquito species poses a major challenge, overshadowing the known vector species *An. minimus* and *An. baimaii*. This finding suggests that *An. culicifacies* has a high potential to emerge as a vector in this region [26, 50, 51].

A previous study conducted in Madhya Pradesh, Gujarat, Karnataka, and Maharashtra recorded a human blood index (HBI) of *An. culicifacies* ranging from 0.02 to 0.22 [29]. Our study recorded a similar range for HBI (0.01–0.34) for this species, with the highest value of 0.34 in Kokrajhar, Assam. The indoor trap density of *An. culicifacies* was lower than the outdoor trap density at most of the study sites, except in the Dahod, Surendranagar, and South Tripura districts. This finding suggests that this species may exhibit mixed host-seeking behavior and varied resting patterns. Hand-catches of resting *An. culicifacies* indicated that the per-man-hour density was higher outdoors in Bareilly, Kokrajhar, and Udalgiri districts, with the highest density (MHD = 27.16) recorded in Bareilly, Uttar Pradesh. The fed-to-half-gravid/gravid ratios of *An. culicifacies* in pyrethrum spray sheet (PSC) collections were greater than 1 at most of the study sites. Both hand catches of resting mosquitoes and PSC results indicated that the resting tendency of *An. culicifacies* are not uniform across the country [29].

*An. baimaii* and *An. minimus* exhibited contrasting resting behavior at the study sites of the northeastern states. *An. baimaii* was predominantly exophilic in both Assam and Tripura [14, 16]. While *An. minimus* was endophilic in Assam, it exhibited exophilic behavior in Tripura, indicating a need for further investigation to understand the shift in resting behavior [16, 52]. The exophilic behavior of this species might be a response to the high usage of chemical-based interventions, such as indoor residual spraying (IRS) and long-lasting insecticidal nets (LLINs). Anopheline mosquitoes adapt their behavior in different environment, and changing their resting patterns is a strategy to avoid insecticide exposure. A transition from endophily to exophily due to the large-scale introduction of LLINs has been documented in *An. fluviatilis* in Odisha [53]. Similar changes were observed for *An. gambiae* s.s. in Tanzania and *An. funestus* in Kenya [54].

In the case of *An. stephensi*, the indoor resting density was higher than the outdoor density at all the study districts, indicating the endophilic behavior of this species. The DE of this species, calculated from the PSC in all the study areas, did not suggest any significant exophilic behavioral change. A similar observation was made in Côte d’Ivoire, where, despite the use of long-lasting insecticide-treated nets (LLINs), 46% of blood-fed *An. gambiae* were found in PSC, and a high proportion of mosquitoes rested indoors [55]. In human landing collections, the density of *An. culicifacies* was higher indoors, indicating an indoor biting preference of this species. In contrast, the lower indoor landing density of *An. stephensi* compared with outdoors suggest its outdoor biting preference in Goa, and this change in biting behavior might be owing to the use of LLINs indoors during biting hours [56–58]. Furthermore, the indoor trap density of *An. stephensi* in Tamil Nadu was found to be 0.25 in this study, and no specimen was found in the outdoor collection. Similar findings was noted in the previous studies which reports 10% more of *An. stephensi* compared to the outdoors of the human dwellings [59].

*An. stephensi* is also a cause for concern because of its invasive behavior. It is distributed in most states in the country, and bionomics studies have been conducted only in a few regions, such as Chennai, Goa, Delhi, Haryana, and Kolkata [60–63]. *An. stephensi* is a major vector in urban areas and can transmit malaria at low densities, whereas it is a poor vector in rural areas [9]. In many urbanized areas of India, the type form of *An. stephensi* is the primary vector of disease transmission, and its abundance greatly exceeds that of mysorensis and intermediate forms [9, 19]. In the absence of bovine blood, *An. stephensi* strongly preferred to feed on humans and thus was a major contributor to urban malaria transmission [19]. In Rajasthan, this species breeds profusely during monsoon and post-monsoon seasons and triggers malaria transmission along with *An. culicifacies* [64]. Compared with other study sites, *An. stephensi* had a higher human blood index (0.62) in Barmer, Rajasthan. A previous study conducted in the Bikaner district showed that more than 50% of *An. stephensi* prefer humans to obtain blood meals [65]. A similar result was found in another study from Jaisalmer district, where blood meal analysis showed that *An. stephensi* had a greater preference for humans (58.2%) [66]. *An. stephensi* host-preference behavior reported in both of these studies is aligned with our findings. In our study, blood meal analysis revealed a high HBI of *An. baimaii* (0.82) and *An. minimus* (0.94), indicating their higher anthropophagic behavior in Northeast India, which is in agreement with previous studies [16, 29].

In this study, we examined 3673 anopheline mosquitoes for *Plasmodium* infection, of which 20 specimens of different vector species tested positive for *P. falciparum* (13) and *P. vivax* (7) (Table 4). A nationwide vector surveillance study (2017–2020) reported a high sporozoite rate of 4.24% in *An. culicifacies* from Haryana State [29]. In our study, a comparatively lower sporozoite rate was found in this species from Dahod district in Gujarat (1%). Another study conducted (2015–2016) in Haryana State reported a sporozoite rate of 0.26% for *An. culicifacies* [67]. In addition, a report from the Kandhamal district in Odisha showed a sporozoite rate of 1.39% in *An. culicifacies* [10]. These results indicate that *An. culicifacies* infection load is uneven throughout the country. Among all the *An. stephensi* samples collected from different study sites, only in Goa the species was found with sporozoite rates of 1.75% and 1.1% in North and South Goa districts, respectively (Table 4). These findings can be correlated with the burden of migrant malaria cases in this state attributed to the influx of workers and tourists from different parts of the country [68].

In the current study, *An. culicifacies* was found resistant to DDT (4%) in Chhattisgarh, Uttar Pradesh, and Tripura. Previous studies have reported resistance to DDT in this species from states such as Odisha, Madhya Pradesh, Jharkhand, Gujarat, Uttar Pradesh, and northeastern states [21, 29]. In addition, resistance to deltamethrin (0.05%) was observed in *An. culicifacies* at Gujarat study sites. There have been earlier reports of deltamethrin resistance (0.05%) in *An. culicifacies* from Odisha, Jharkhand, Maharashtra, Karnataka, Gujarat, and Haryana [21, 27, 69–71]. Furthermore, *An. culicifacies* developed resistance to alpha-cypermethrin (0.05%) in Chhattisgarh and to malathion (5%) in Uttar Pradesh, Tripura, and Chhattisgarh, thus posing a significant challenge to the current ongoing intervention strategies. An earlier study reported that *An. stephensi* was resistant to malathion, DDT, and deltamethrin in Haryana [21, 27]. Recently, triple insecticide resistance in *An. stephensi* has been reported for the first time [29]. In the current study, *An. stephensi* was found to be resistant to DDT and malathion, while it showed possible resistance to deltamethrin, with 91–94% mortality. Indoor residual spraying (IRS) and long-lasting insecticidal nets (LLINs) are primary vector control interventions in India where synthetic pyrethroids are used [2]. The emergence of resistance to deltamethrin, a synthetic pyrethroid, is a significant concern for intervention perspective [21, 72]. Insecticide resistance management aims to prevent and to delay the development of resistance in mosquito populations. To develop effective usage and resistance management techniques, it is essential to understand the mechanisms of resistance in malaria vectors in various ecological contexts. Further, research is needed to evaluate the frequency and intensity of insecticide resistance in malaria vectors, as well as the underlying mechanisms, to develop effective resistance management strategies [73]. In addition, insecticide resistance management strategies must be designed and deployed in areas where vectors have developed resistance to multiple classes of insecticides. For instance, a long-lasting insecticidal net (LLIN) incorporating piperonyl butoxide and alphacypermethrin, evaluated by the Indian Council of Medical Research-Vector Control Research Centre (ICMR-VCRC) in Odisha, suggesting its use in limited areas [74]. The rotation of insecticides might help to delay resistance or regain susceptibility to commonly used insecticides in public health programs. There is a need to consider newer tools and strategies for vector control that can help accelerate malaria elimination efforts in India [25].

## Conclusions

This study provides valuable insights into the relative abundance of primary vector species, their biting behavior, host preferences, resting patterns, infectivity rates, and susceptibility to currently used insecticides. Understanding these factors will enhance vector control efforts and assess their effectiveness.

Our findings confirm that *An. culicifacies* is the primary vector in central, northern, and western states such as Chhattisgarh, Uttar Pradesh, and Gujarat. *An. minimus* and *An. baimaii* are the vectors transmitting malaria in the northeastern states of India. In addition, *An. stephensi* continues to be responsible for malaria transmission in the western coastal state of India.

This study also indicated a change in resting behavior of *An. minimus* in Tripura from endophilic to exophilic, which would be a major concern. This underscores the importance of ongoing surveillance monitoring of this species to better understand its distribution in these regions. In addition, our findings highlight the increasing insecticide resistance of both *An. culicifacies* and *An. stephensi* to pyrethroids, posing a serious threat to ongoing vector control programs. This study also provides updated data on the distribution of primary vector species, which can be used to suggest appropriate, situation-specific vector control strategies.

The study was envisaged to generate data on malaria vector bionomics and insecticide susceptibility following a standard protocol across multiple endemic states; however, a few logistical and operational limitations were encountered. Certain indicators, such as outdoor resting collections, human biting rate assessments, and abdominal condition observations could not be uniformly captured across all sites owing to constraints in field conditions and resource availability. Insecticide resistance tests could not be performed for all insecticides because of limited numbers of mosquitoes and material supplies, and data collection frequency varied in some districts. These constraints were carefully considered during data analysis, and the results were interpreted with appropriate caution. Despite these limitations, the study offers robust and comprehensive insights into malaria vector bionomics across diverse ecological settings in India.

## Data Availability

All data supporting the findings and conclusions of this study are included within the manuscript.
